# Genome-Wide Identification and Expression Pattern Analysis of GATA Gene Family in Orchidaceae

**DOI:** 10.3390/genes15070915

**Published:** 2024-07-13

**Authors:** Qinyao Zheng, Ye Huang, Xin He, Meng-Meng Zhang, Zhong-Jian Liu

**Affiliations:** 1 Key Laboratory of National Forestry and Grassland Administration for Orchid Conservation and Utilization at College of Landscape Architecture, Fujian Agriculture and Forestry University, Fuzhou 350002, China; 2 College of Forestry, Fujian Agriculture and Forestry University, Fuzhou 350002, China

**Keywords:** floral development, heat stress, abiotic stress

## Abstract

The GATA transcription factors play crucial roles in plant growth, development, and responses to environmental stress. Despite extensive studies of GATA genes in many plants, their specific functions and mechanisms in orchids remain unexplored. In our study, a total of 149 GATA genes were identified in the genomes of seven sequenced orchid species (20 *PeqGATAs*, 23 *CgGATAs*, 24 *CeGATAs*, 23 *DcaGATAs*, 20 *DchGATAs*, 27 *DnoGATAs*, and 12 *GelGATAs*), classified into four subfamilies. Subfamily I typically contains genes with two exons, while subfamily II contains genes with two or three exons. Most members of subfamilies III and IV have seven or eight exons, with longer introns compared to subfamilies I and II. In total, 24 pairs (*CgGATAs*–*DchGATAs*), 27 pairs (*DchGATAs*–*DnoGATAs*), and 14 pairs (*DnoGATAs*–*GelGATAs*) of collinear relationships were identified. Cis-acting elements in GATA promoters were mainly enriched in abscisic acid (ABA) response elements and methyl jasmonate (MeJA) elements. Expression patterns and RT-qPCR analysis revealed that GATAs are involved in the regulation of floral development in orchids. Furthermore, under high-temperature treatment, *GL17420* showed an initial increase followed by a decrease, *GL18180* and *GL17341* exhibited a downregulation followed by upregulation and then a decrease, while *GL30286* and *GL20810* displayed an initial increase followed by slight inhibition and then another increase, indicating diverse regulatory mechanisms of different GATA genes under heat stress. This study explores the function of GATA genes in orchids, providing a theoretical basis and potential genetic resources for orchid breeding and stress resistance improvement.

## 1. Introduction

GATA proteins are a group of transcription factors capable of specifically recognizing and binding to the WGATAR (W = T or A; R = G or A) consensus sequence, found in a variety of organisms including plants, fungi, nematodes, and insects [1]. Despite structural differences in GATA proteins among different species, most GATA transcription factors in plants contain one or two highly conserved zinc-finger DNA-binding domains, commonly with the sequence CX₂CX₁₈–₂₀CX₂C (type IV zinc-finger domain) [2,3]. Based on evolutionary relationships and gene structure analysis, the GATA gene family can be divided into four subfamilies, each with distinct differences in the number of exons [4]. This exon–intron pattern and conserved zinc-finger domain highlight the evolutionary conservation and diversification of GATA transcription factors across different plant species, underscoring their crucial roles in plant development and stress responses.

As an important group of regulatory proteins, the GATA gene family has been reported to play a significant role in regulating flower development and responding to abiotic stresses such as drought and salt stress. In terms of flower development, many functions associated with GATA genes have been validated in the model plant *Arabidopsis thaliana*. For instance, members of the GATA3 family, HANL2 and GNL, primarily participate in the regulation of sepal separation and petal number, while GNC is involved in sepal separation, petal number, and the development of stamens and carpels [5]. Additionally, the GATA3 family functions redundantly with the key transcriptional repressor HANABA TARANU, regulating genes involved in hormone signaling and floral organ specification to control flower development [5,6]. The paralogs and functionally redundant GATA transcription factors *AtGNC* and *AtGNL*/*CGA1* control flowering time by repressing the transcription of *SOC1* [7]. Moreover, *AtGNC* and *AtGNL* are negatively regulated by AP3/PI to promote proper floral organ type differentiation and development [8]. ZIM (GATA-1-type, putative single zinc finger), also known as GATA25, has been identified to be expressed in the shoot apex and flowers during the reproductive stage of *Arabidopsis*, which is involved in the development of inflorescences and flowers [9], and plays a role in accelerating flowering time under long-day conditions [10]. GATA transcription factors are essential not only for the floral organs of model plants but also for the flower development of other plants, including apples [3], wheat [11], and *Brachypodium distachyon* [12]. Regarding abiotic stress, previous studies have reported that *IbGATA24* in sweet potatoes positively regulates tolerance to drought and salt stress through interaction with *IbCOP9-5a* [13]. Additionally, *OsGATA16* in rice enhances cold tolerance by repressing *OsWRKY45-1* during the seedling stage [14]. *PdGNC* confers drought tolerance by mediating stomatal closure in *Populus deltoides* [15]. The expression patterns of GATA subfamily I members in tomatoes under abiotic stress indicate responses to cold, drought, and salt stresses [16].

Orchidaceae is one of the largest groups of angiosperms, comprising over 750 genera and more than 29,000 species [17]. Due to their rich ornamental value, orchids are in high demand in the horticultural market, forming a vast industrial chain [18]. However, various abiotic stresses have impacted the growth and development of orchids in recent years. These environmental challenges not only hinder the physiological processes essential for orchid survival but also reduce their flowering potential and aesthetic value, thereby affecting their economic viability and conservation [19]. Therefore, understanding the molecular mechanisms of flower development and heat response in orchids is crucial for improving their stress resistance and breeding effectiveness. The GATA gene family plays a significant role in enhancing plant stress resistance and breeding, but to date, no research has focused on GATA genes in orchids. So, our study is the first to comprehensively identify the GATA gene family in Orchidaceae, systematically analyzing phylogenetic relationships, physicochemical properties, chromosomal localization, gene structure, motif composition, collinearity, and promoter characteristics. Additionally, we used qRT-PCR to examine the expression patterns of *CgGATAs* under heat treatment to explore their functions and mechanisms. We believe this research provides new insights into the study of stress resistance in orchids and offers an important theoretical foundation for future orchid breeding and improvement efforts. By elucidating the molecular mechanisms underlying stress responses, this knowledge can be applied to enhance the resilience of orchids, particularly in their ability to withstand environmental challenges, thereby contributing to the sustainability and economic viability of orchid cultivation.

## 2. Materials and Methods

### 2.1. Plant Materials and Data Sources

The plant materials, *Cymbidium goeringii*, were obtained from Fujian Agriculture and Forestry University in Fujian, China. We selected three pots of *C. goeringii* with similar growth conditions and subjected them to heat stress in a controlled environment chamber. The heat stress was applied under the following conditions: 16 h of light at 30 °C followed by 8 h of darkness at 38 °C. The samples were subjected to heat stress (40 °C) for 0 h, 6 h, 12 h, and 18 h, respectively, while keeping other conditions constant. Leaf samples from each group were collected at the corresponding time points and rapidly frozen in liquid nitrogen for subsequent experiments. In addition, the whole-genome sequences and annotation files of seven orchid species were downloaded from NCBI (https://www.ncbi.nlm.nih.gov/, accessed on 19 November 2023) and the National Genomics Data Center (NGDC) (https://ngdc.cncb.ac.cn/, accessed on 19 November 2023), with the accession numbers as follows: *Phalaenopsis equestris* (PRJNA192198) [20], *C. goeringii* (PRJNA749652) [21], *C. ensifolium* (PRJCA005355) [22], *Dendrobium catenatum* (PRJNA262478) [23], *D. chrysotoxum* (PRJNA664445) [24], *D. nobile* (PRJNA725550) [25], and *Gastrodia elata* (PVEL00000000) [26]. The GATA protein sequence of *A. thaliana* was obtained from TAIR (https://www.arabidopsis.org/, accessed on 25 November 2023).

### 2.2. Identification, Physicochemical Properties and Phylogenetic Tree

Using 30 *AthGATA* sequences as query sequences and setting the e-value to 1 × 10^−5^, we employed the Blast tool and Simple HMM Search (PF00320) of TBtools v2.096 [27] to search and identify potential candidate GATA genes in seven orchid genomes, including *P. equestris*, *C. goeringii*, *C. ensifolium*, *D. catenatum*, *D. chrysotoxum*, *D. nobile*, and *G. elata*. Through conservative structural filtering, incomplete or redundant protein sequences were manually removed. Additionally, the physicochemical properties analysis was conducted using the online software ExPASy 3.0 (https://www.expasy.org/, accessed on 28 January 2024). Through orchid protein sequences calculations, we obtained results for amino acid composition (AA), isoelectric point (pI), molecular weight (MW), grand average of hydropathicity (GRAVY), instability index (II), and aliphatic index (AI). Additionally, we inputted a total of 179 protein sequences into PhyloSuite v1.2.3 [28] and conducted alignment using MAFFT [29] with default parameters. Subsequently, we constructed a neighbor-joining (NJ) phylogenetic tree of GATAs with a bootstrap value of 1000 and a minimum correlation coefficient set to 0.90. The online software Evolview 3.0 (http://www.evolgenius.info/evolview/#/treeview, accessed on 8 February 2024) was used for visualization.

### 2.3. Chromosomal Localization

We imported the GFF files of each orchid genome into TBtools v2.096 [27] to generate the chromosomal location map for each GATA gene.

### 2.4. Gene Structure and Multiple Sequence Alignment

Using the online tool MEME (https://meme-suite.org/meme/tools/meme, accessed on 12 February 2024), we predicted the conserved motifs of GATA genes in seven orchid species. The maximum number of motifs was set to ten, with other parameters kept at default settings. The MAST file was downloaded for gene structure visualization and analyzed using TBtools v2.096 [27]. The multiple sequence alignment was also visualized in TBtools v2.096 [27] with the aligned protein sequences.

### 2.5. Synteny Analysis and Cis-Regulatory Element Analysis

Using One Step MCScanX-SuperFast in TBtools v2.096 [27], we identified the collinearity relationships among *C. goeringii*, *D. nobile*, *D. chrysotoxum*, and *G. elata*. The resulting ctl, collinearity, and GFF files were input into the Dual Synteny Plot function in TBtools v2.096 [27] to generate the visualization. To predict cis-acting elements in four orchid species, we extracted the upstream sequences (2000 bp) of GATA genes using GFF files and analyzed these sequences on the PlantCARE website (https://bioinformatics.psb.ugent.be/webtools/plantcare/html/, accessed on 17 February 2022). We excluded common promoter elements and retained those related to stress resistance. The manually curated results were then visualized using TBtools v2.096 [27].

### 2.6. Expression Analysis and RT-qPCR

To study the expression patterns of GATAs in different floral parts of orchids, we first performed quality control on the raw RNA-seq data using fastp [30]. The processed RNA-seq reads were then used for transcript quantification, converting the expression levels of each gene to fragments per kilobase of transcript per million mapped reads (FPKM). We established an RNA-seq transcriptome database for different floral parts at various stages, with three biological replicates for each sample. Finally, the heatmap was generated in TBtools v2.096 [27] based on the FPKM values to visualize the expression of GATA genes at different floral parts.

The total RNA of *C. goeringii* was extracted using the FastPure Plant Total RNA Isolation Kit (for polysaccharide- and polyphenol-rich tissues) (Vazyme Biotech Co., Ltd., Nanjing, China) according to the manufacturer’s instructions. The extracted RNA was then reverse transcribed into cDNA using Hifair^®^ AdvanceFast One-step RT-gDNA Digestion SuperMix for qPCR (Yeasen Biotechnology Co., Ltd., Shanghai, China). The cDNA was then used as a template for qPCR with Hieff^®^ qPCR SYBR Green Master Mix (High Rox Plus). Primers specific to the target gene and qPCR dye were added, and the analysis was conducted on an ABI 7500 Real-Time System. The primers were designed using Primer Premier 5 software. The RT-qPCR conditions were 5 min at 95 °C for the pre-denaturation process, followed by 40 cycles of 10 s at 95 °C and 30 s at 60 °C during the amplification phase (the cycling stage). The experimental setup utilized 96-well plates with a 20 μL reaction system in each well, and 3 biological replicates were performed in this study. The data obtained were processed using the 2−ΔΔCT method and visualized using GraphPad Prism 7.0.

## 3. Results

### 3.1. Phylogenetic Analysis of GATA Genes

**Figure 1:**
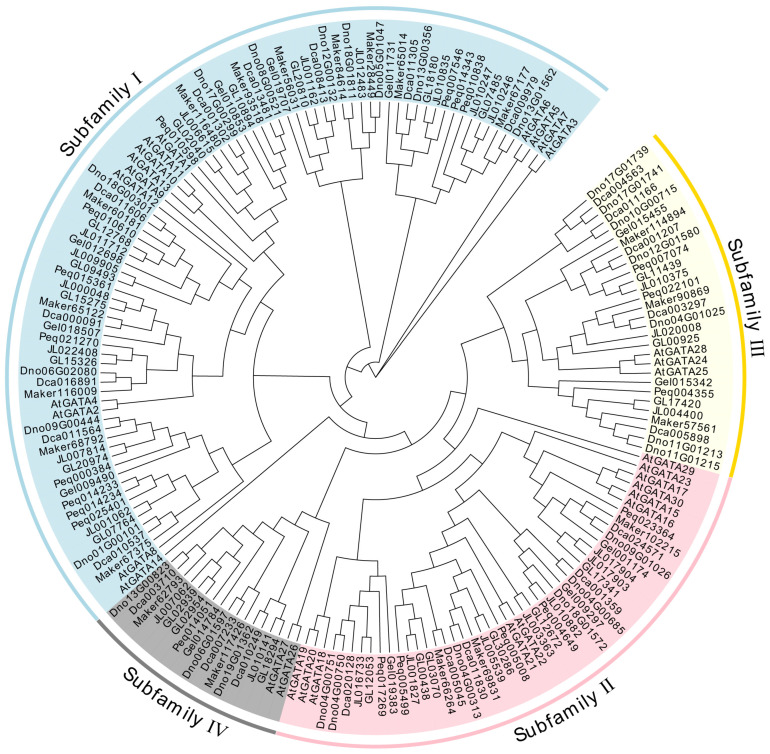
Phylogenetic tree of GATA genes from seven orchid species and *A. thaliana*.

As illustrated in Figure 1, a total of 179 GATA genes were classified into 4 subfamilies, designated as subfamily I, II, III, and IV. Subfamily I contains the largest number of members, with 87 genes, followed by subfamily II with 46 genes, subfamily III with 29 genes, and subfamily IV with 17 genes, the fewest. The majority of orchid GATA genes are concentrated in subfamilies I and II. Notably, a significant proportion of GATA members in subfamily III are from *D. catenatum* and *D. nobile*, with five and seven members, respectively.

### 3.2. Identification and Physicochemical Properties of the GATA Gene Family

**Figure 2:**
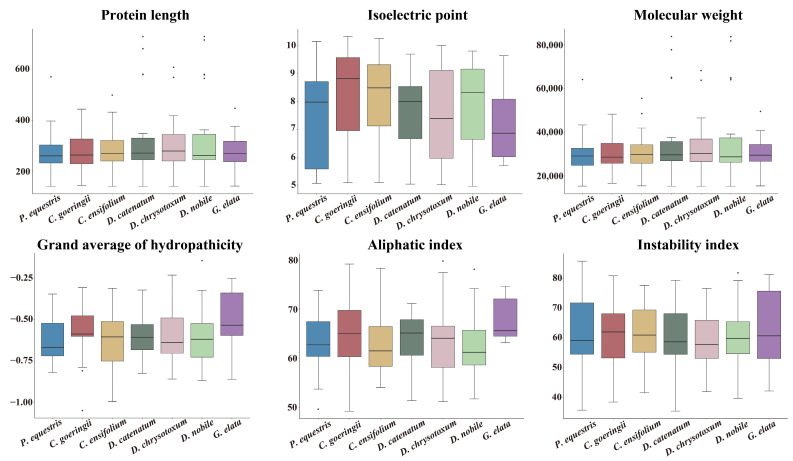
Physicochemical properties of orchid GATA proteins.

Using the *Arabidopsis* GATA protein sequences as a reference, we identified a total of 149 GATA genes across seven orchid species. Specifically, there were 20 in *P. equestris*, 23 in *C. goeringii*, 24 in *C. ensifolium*, 23 in *D. catenatum*, 20 in *D. chrysotoxum*, 27 in *D. nobile*, and 12 in *G. elata*. To further understand the functions and mechanisms of these GATA proteins, we predicted and analyzed their physicochemical properties (Figure 2). These properties included protein length, isoelectric point, molecular weight, grand average of hydropathicity (GRAVY), aliphatic index (AI), and instability index (II). Our results revealed that protein lengths ranged from 140 amino acids (aa) to 730 aa, with most being around 270 aa. The isoelectric points ranged from 4.97 (*Dno06G02080*) to 10.33 (*GL03070*), with 56 GATA proteins being acidic (isoelectric point below 7) and 93 being basic (isoelectric point above 7). The molecular weights ranged from 15,276.37 Da (*Dno09G01026*) to 83,833.85 Da (*Dca004563*), with an average molecular weight of 33,301.98 Da. All GATA proteins had GRAVY values less than 0, with the lowest being −1.051 (*GL02040*), indicating that they are hydrophilic proteins. The AI values ranged from 49.17 (*GL02040*) to 79.78 (*Maker67177*), and the II values ranged from 35.52 (*Dca001207*) to 85.84 (*Peq010598*). Detailed physicochemical properties of the related GATA genes are provided in Supplementary Table S1.

### 3.3. Chromosomal Localization of GATA Genes

**Figure 3:**
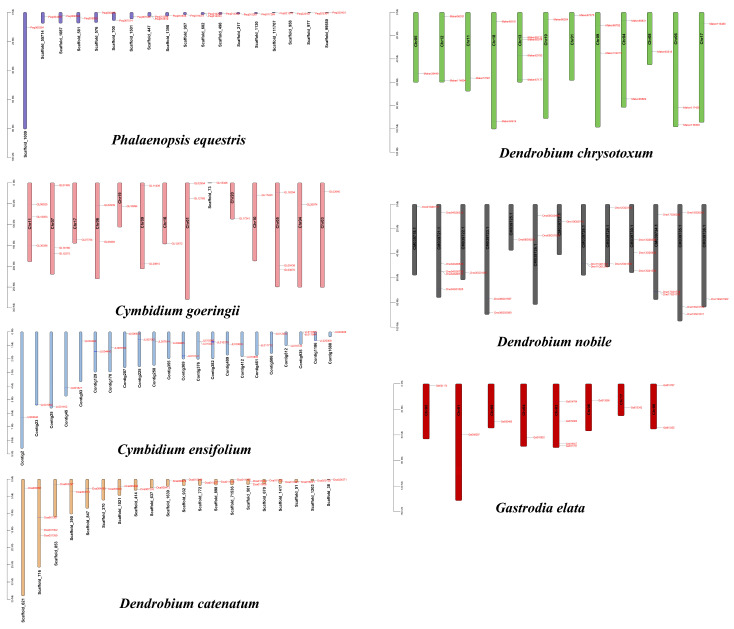
Chromosomal distribution of GATA genes in seven orchid species.

As shown in Figure 3, we found that GATA genes are unevenly distributed across the chromosomes. Specifically, we identified one pair of tandemly duplicated genes on *P. equestris* (*Peq014233*/*Peq014234*), two pairs on *C. ensifolium* (*JL010246*/*JL010247* and *JL017903*/*JL017904*), and one pair on *D. nobile* (*Dno04G00750*/*Dno04G00751*).

### 3.4. Analysis of GATA Gene Structure and Motifs

**Figure 4:**
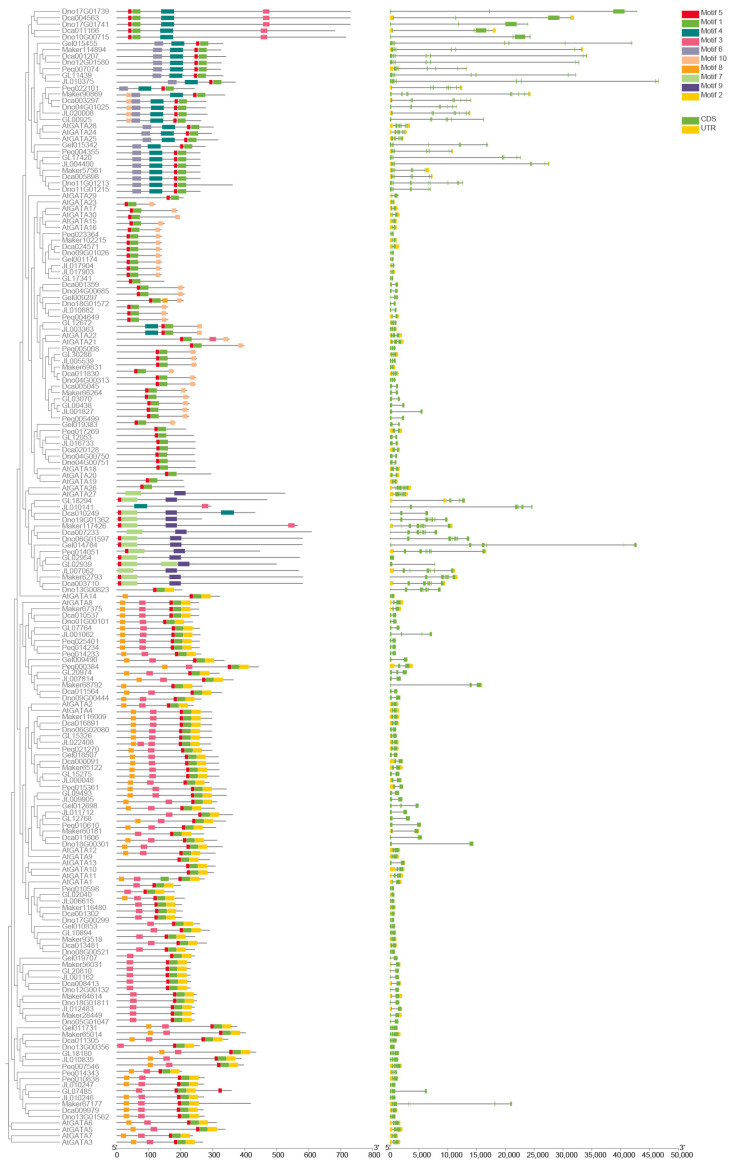
GATA phylogenetic tree, motifs, and structure.

**Figure 5:**
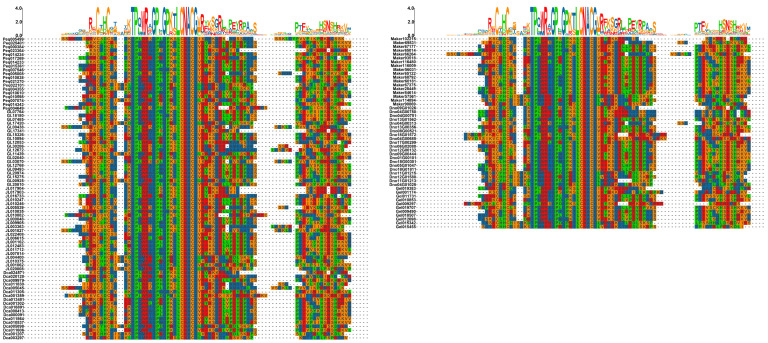
Multiple sequence alignment results of GATA gene family.

To better understand the gene structure characteristics of the GATA gene family in orchids, we predicted multiple conserved motifs and analyzed the distribution of introns and exons within their sequences (Figure 4). Our results clearly show distinct patterns among the subfamilies. Members of subfamily III predominantly display the motif order of motif6–motif4–motif5–motif1, with motif6 being unique to this subfamily and motifs 4, 5, and 1 highly conserved within it. Subfamily II members mostly follow the motif order of motif5–motif1–motif10, with an overall sequence length averaging around 200–300 bp. In subfamily IV, the motif order is motif5–motif7–motif9, with motifs 7 and 9 being unique to this subfamily. Subfamily I members are characterized by the motif order of motif8–motif3–motif5–motif1–motif2, with motifs 8, 3, and 2 being unique and highly conserved within this subfamily. Additionally, the 149 orchid GATA genes contain between one and eight CDS regions. For example, *Dno13G00356* has only one CDS, while most members of subfamilies III and IV contain seven or eight CDS regions. Through multiple sequence alignments of 149 GATA genes, we found that all sequences contain a specific domain (Figure 5), namely the CX_2_CX_18_CX_2_C sequence, which is highly conserved. This domain had several significantly conserved amino acid residues, including cysteine residues (C), glycine residues (G), proline residues (P), asparagine residues (N), and alanine residues (A).

### 3.5. Collinearity Analysis of the GATA Gene Family

**Figure 6:**
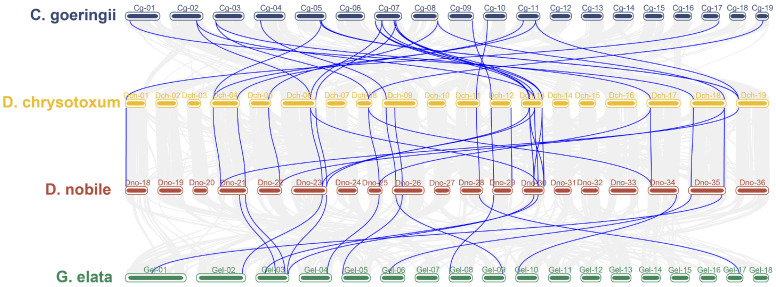
Collinearity relationships among four orchid species.

To identify duplication events of the GATA gene family in orchids, we conducted collinearity analysis on *C. goeringii*, *D. chrysotoxum*, *D. nobile*, and *G. elata*. As shown in Figure 6, there is evidence of collinearity among the GATA genes in these four orchid species, indicating a high level of homology. We identified 24 pairs of collinear relationships between *C. goeringii* and *D. chrysotoxum*, 27 pairs between *D. chrysotoxum* and *D. nobile*, and 14 pairs between *D. nobile* and *G. elata*. Notably, collinear relationships on chromosomes Cg07, Dch-13, Dno-30, and Gel-03 are more abundant than on other chromosomes within the same species. However, the GATA genes in *G. elata* are notably fewer compared to other orchid species.

### 3.6. Cis-Element Analysis

**Figure 7:**
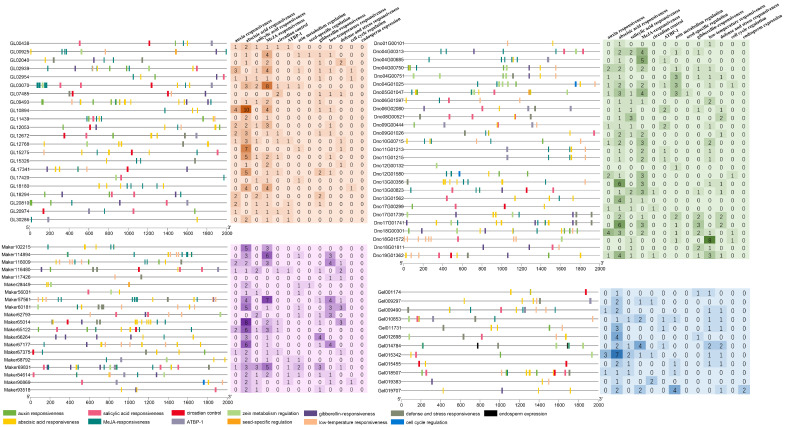
Regulatory elements in the promoter regions of four orchid species. Orange represents *C. goeringii*, purple represents *D. chrysotoxum*, green represents *D. nobile*, and blue represents *G. elata*.

To further investigate the regulatory functions of GATA in orchids, we identified the major cis elements within the 2000 bp upstream regions of the promoters of 4 orchid species, totaling 13 kinds of elements. As shown in Figure 7, the prominent cis elements in the upstream promoters of orchid GATA genes are abscisic acid responsiveness (190) and MeJA responsiveness (134). Notably, apart from these two elements, gibberellin responsiveness, low-temperature responsiveness, and defense and stress responsiveness also occur frequently in *D. chrysotoxum*. In the promoter elements of *D. nobile*, the low-temperature responsiveness element of gene *Dno18G01572* appears eight times. In the promoter elements of *G. elata*, the zein metabolism regulation element of gene *Gel19707* appears four times.

### 3.7. Heatmap Analysis of Floral Components and Heat-Resistant RT-qPCR Analysis

**Figure 8:**
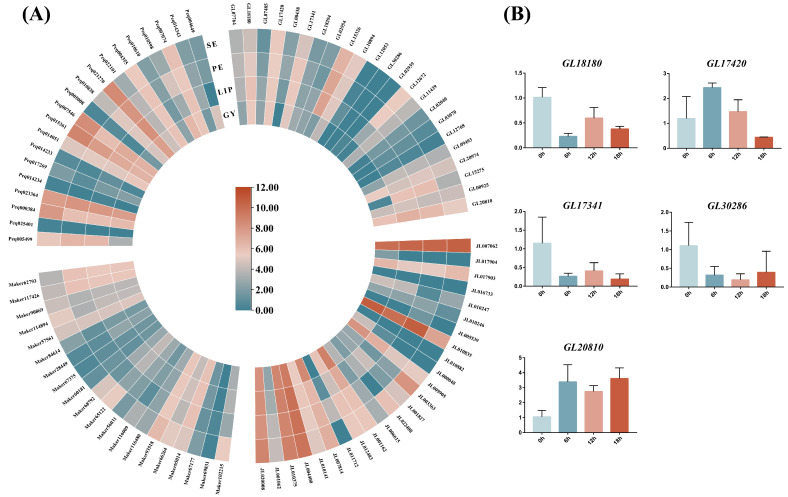
(**A**) Heatmap of expression patterns of the GATA gene family in the floral components of four orchid species. Se: sepal; Pe: petal; Lip: lip; Gy: gynostemium. (**B**) Expression analysis of five *CgGATA* genes in leaves under heat treatment at different time points.

To understand the expression differences of the GATA gene family in different floral components, we compared the expression levels of GATA genes in four orchids. As shown in Figure 8A, there are 7 genes in *C. goeringii* (7/23), 13 genes in *C. ensifolium* (13/24), five genes in *D. chrysotoxum* (5/20), and 10 genes in *P. equestris* (10/20) with high expression in floral organs. Notably, in *C. goeringii*, *GL15326* exhibits high expression levels in the sepal, petal, and lip, but is barely expressed in the gynostemium. *GL12672* shows high expression in the sepal and petal, while *GL15275* and *GL20810* demonstrate high expression in the petal and lip. *JL010835* exhibits greatly higher expression in the petal, lip, and gynostemium compared to the sepal of *C. ensifolium*. *JL009905* shows elevated expression in lip and gynostemium relative to sepal and petal. *JL003363* and *JL022408* are highly expressed exclusively in sepal. In *D. chrysotoxum*, *Maker102215* is predominantly expressed in sepal, whereas *Maker117426* and *Maker62793* show high expression in the other three floral components. *Peq000384*, *Peq015361*, and *Peq007546* display elevated expression in the sepal, while *Peq010598* is highly expressed only in the gynostemium.

To further elucidate the expression patterns of GATA genes in *C. goeringii* under high-temperature stress, we selected five genes for qPCR experiments based on their subfamily classification, the number of promoters, and their expression levels of leaves. The results of the high-temperature treatment (Figure 8B) reveal three distinct patterns. The first pattern is exhibited by *GL17420*, whose expression sharply increases within 6 h and then gradually decreases over time. The second pattern is shown by *GL18180* and *GL17341*, both of which display a significant downregulation after 6 h of high-temperature treatment, followed by a rapid upregulation at 12 h, and a subsequent decrease in expression at 18 h. The third pattern, opposite to the second, is observed in *GL30286* and *GL20810*, which show upregulation at 6 h, slight repression at 12 h, and further upregulation at 18 h, with expression levels at 18 h being higher than those at 6 h.

## 4. Discussion

The transcription factor GATA has been extensively studied in many plants and animals. GATA genes participate in various critical biological processes in plants by regulating genes responsible for the development of different tissues and modulating hormone signaling under stress conditions [6]. However, the function and molecular mechanisms of the GATA gene in regulating different floral components and heat tolerance have not yet been explored in orchids. Therefore, in this study, we identified the GATA gene family in orchids for the first time. A total of 149 GATA genes were identified in orchids, including 20 in *P. equestris*, 23 in *C. goeringii*, 24 in *C. ensifolium*, 23 in *D. catenatum*, 20 in *D. chrysotoxum*, 27 in *D. nobile*, and 12 in *G. elata*. The number is comparable to the GATA genes in the monocot *Oryza sativa* [2], but fewer than in other monocots, such as wheat [31], maize [32,33], and *Sorghum bicolor* [34]. The highest number of GATA family members, 96 genes, has been found in *Brassica napus* [35], emphasizing the variability in gene family size influenced by genome size, chromosome number, and gene duplication events [36]. A phylogenetic analysis comparing the 149 GATA genes in orchids with those in *A. thaliana* revealed that the distribution of GATA genes across different subfamilies in orchids is relatively conserved. Our results indicate that the largest number of members in orchids is found in subfamily I, followed by subfamily II, with subfamily IV having the fewest members. This pattern is similar to the phylogenetic classification observed in apple [3], *B. distachyon* [12], and grape [37], where subfamily I also has the most members and subfamily IV the fewest. Additionally, the phylogenetic analysis indicates that the number of genes in subfamily III is significantly higher in *D. catenatum* and *D. nobile* compared to other orchid species, possibly due to gene duplication events within this subfamily in *Dendrobium* species. The physicochemical properties results indicate that the instability index of most GATA proteins (145 out of 149) is greater than 40.00, suggesting that they are unstable proteins [38]. All GATA proteins have negative GRAVY values (−0.142 to −1.051), identifying them as hydrophilic proteins. Based on their isoelectric points, the ratio of acidic to basic proteins is approximately 2:3 (56:93). Our results revealed that protein lengths ranged from 140 amino acids (aa) to 730 aa in orchids, whereas GATA factors in soybean encode peptides ranging from 80 to 551 aa [39]. In wheat, *TaGATA* proteins have lengths ranging from 146 to 499 aa [31], and in *B. napus*, GATA proteins range from 101 to 576 aa [35]. This variation in protein length suggests that the GATA gene family exhibits considerable diversity in different species, which may be attributed to species-specific evolutionary adaptations and functional requirements. Additionally, this diversity in amino acid length could influence the stability and functionality of the proteins. Understanding these differences in protein length can provide insights into the evolutionary pressures and functional diversification of GATA proteins across various plant species.

The GATA gene structure analysis results (Figure 4) reveal that each subfamily contains unique motifs and conserved motif sequences. Notably, motif8, motif3, and motif2 are specific to subfamily I, motif7 and motif9 are exclusive to subfamily IV, and motif6 is distinctive to subfamily III. These findings suggest that each subfamily may possess certain specialized functions, distinguishing them from other subfamilies. In *A. thaliana*, subfamily I of the GATA family comprises 14 members, each with 2 exons. Subfamily II includes ten members with two to three exons, while subfamily III consists of three members, each containing seven exons. Subfamily IV lacks characteristic gene structure features [2]. Our results reveal that in orchids, subfamily I includes 73 members with 2 exons (73/87). Subfamily II consists of 31 members with 2 exons (31/46), with the remaining 14 members having 3 exons. Most members of subfamilies III and IV have seven or eight CDS. This clearly indicates that the exon distribution in orchid GATA subfamilies I, II, and III is similar to that in *A. thaliana*. However, subfamily IV in orchids distinctly features seven exons. Additionally, the pattern of having fewer introns in subfamilies I and II and a higher number of introns in subfamilies III and IV is also observed in *Fagopyrum tataricum* [40] and *Eucalyptus urophylla* [41]. This suggests a conserved evolutionary mechanism influencing exon–intron structure across different species, contributing to the functional diversity of GATA genes. In addition to the exon number, we found that intron lengths in subfamilies III and IV are generally longer than those in subfamilies I and II. During gene evolution, longer introns are often favored as they enhance the efficiency of natural selection by increasing recombination between adjacent exons [42], suggesting that introns may have significant implications for the evolution and functional diversification of the GATA gene family.

Gene duplication is a major driving force behind the expansion of gene families, significantly contributing to novelty and diversification in plants [43,44]. Segmental duplication, tandem duplication, and transposition events are considered crucial mechanisms in plant evolution [45]. Based on the synteny analysis of the GATA gene family in four orchid species and their chromosomal localization, this study identified two pairs of tandemly duplicated genes in *P. equestris* and *C. ensifolium*, namely *Peq014233*/*34* and *JL010246*/*47*, both belonging to subfamily I. Additionally, two pairs of tandemly duplicated genes were identified in *C. ensifolium* and *D. nobile*, namely *JL017903*/*04* and *Dno04G00750*/*51*, both belonging to subfamily II. These findings suggest that these four pairs of genes may co-regulate related biological processes through their transcriptional activities. Additionally, we identified 24 syntenic relationships between *C. goeringii* and *D. chrysotoxum*, 27 between *D. chrysotoxum* and *D. nobile*, and 14 between *D. nobile* and *G. elata*. This comparative analysis indicates that the GATA genes in these orchid species do not correspond one to one, suggesting that duplication events have occurred within the orchid GATA gene family, leading to its expansion. Cis-acting elements are DNA sequences with regulatory activity that control gene expression, playing a crucial role in development and physiology [46]. They ensure the correct spatiotemporal pattern of gene expression, which is essential for proper development and environmental responses [47]. In this study, we predicted the cis-acting elements in the promoter regions of 82 GATA genes, identifying elements associated with growth and development, physiological regulation, abiotic stress, and plant hormones. Our results revealed that abscisic acid (ABA) response elements were the most frequently occurring, followed by methyl jasmonate (MeJA) elements. The plant hormone ABA plays a significant role in helping plants adapt to abiotic environmental stresses [48], while MeJA assists plants in coping with various types of environmental stress, such as salt stress, drought, and low temperatures, thereby enhancing plant resilience and survival through multiple mechanisms. Notably, *Dno18G01572* contains eight low-temperature responsive elements, indicating its high sensitivity to environmental changes and its potential role in regulating various cold adaptation mechanisms. Similarly, six GATA genes in *D. chrysotoxum* are significantly enriched with multiple low-temperature responsive elements. *Gel019707* is not only involved in zein metabolism regulation but also plays a crucial role in endosperm expression. In summary, we suggest that the GATA genes in *Dendrobium* may play a significant role in cold adaptation and environmental stress response. By regulating stress physiology and hormone signal transduction, these genes enhance the plant’s defense capabilities against fluctuating environmental conditions.

The orchid family is one of the largest among flowering plants, with its flowers being essential for the study of plant developmental biology [49]. In *Arabidopsis*, GNC and GNL have been identified as genes that inhibit flowering [50]. In *B. napus*, the expression of the *BnGATA2.5* gene has been linked to flowering time [51]. Previous research on the GATA gene has primarily focused on its influence on flowering time regulation or organ abscission, whereas detailed studies on GATA expression in various floral parts are relatively scarce. By integrating transcriptome data from different floral parts of four orchid species, our study reveals that GATA genes exhibit significant tissue-specific expression. Specifically, In *C. goeringii*, *C. ensifolium*, *D. chrysotoxum*, and *P. equestris*, GATA genes exhibit significant tissue-specific expression, with seven, thirteen, five, and ten genes, respectively, showing high expression in floral parts, which indicates the critical regulatory role of GATA genes in floral organ development and provides a crucial basis for further exploration of their specific mechanisms in flower organ formation and functional regulation. Furthermore, the GATA gene family plays an important role in plant responses to abiotic stresses, contributing to enhanced tolerance to low temperatures and drought. Previous studies have indicated that in barley, GATA gene expression levels are markedly upregulated under flooding conditions [52]. In chickpeas (*Cicer arietinum*), under abscisic acid and dehydration stress, these genes may participate in regulating chickpeas’ response to water stress in an abscisic acid-dependent manner [53]. Regarding plant cold resistance, *OsGATA16* exerts a positive regulatory role in enhancing cold resistance during the seedling stage of rice by binding to the promoter of *OsWRKY45-1* and suppressing its expression [14].

Furthermore, our study reveals that in the first pattern, the expression of *GL17420* initially increases and then decreases under high-temperature treatment. This pattern suggests that *GL17420* may play a crucial role in the early stages of high-temperature stress by rapidly responding to heat stimuli, such as through the expression of heat shock proteins, and then gradually adapting as the stress continues. In the second pattern, *GL18180* and *GL17341* are significantly downregulated initially, followed by a rapid upregulation and subsequent decrease under high-temperature treatment. This pattern likely reflects the complex roles of these genes in regulating the response to heat stress. The initial downregulation may be associated with the suppression of non-essential physiological processes to conserve energy, while the later upregulation could be to activate specific defense mechanisms or repair damage. The third pattern (*GL30286* and *GL20810*), which is opposite to the second, involves an initial upregulation, followed by a slight repression, and then a final upregulation. This pattern indicates a sustained positive response of these genes to high-temperature stress. These results suggest that the GATA genes in *C. goeringii* exhibit dynamic, time-dependent expression patterns under heat stress, revealing diverse regulatory mechanisms employed by different GATA genes in response to high-temperature stress.

## 5. Conclusions

We identified a total of 20 *PeqGATAs*, 23 *CgGATAs*, 24 *CeGATAs*, 23 *DcaGATAs*, 20 *DchGATAs*, 27 *DnoGATAs*, and 12 *GelGATAs*, classified into 4 subfamilies. Phylogenetic analysis, gene structure, promoter prediction, chromosomal localization, and functional validation were conducted on the GATA genes from seven orchid species. Our study reveals that subfamilies I and II have fewer and shorter introns, while subfamilies III and IV have more and longer introns. This pattern suggests a conserved evolutionary mechanism, enhancing our understanding of GATA gene structure in orchids. The results revealed significant tissue-specific expression of GATA genes in floral organs and diverse regulatory mechanisms under heat stress. *GL17420* shows a rapid response, while *GL18180* and *GL17341* first downregulate and then upregulate, and *GL30286* and *GL20810* first upregulate, then are slightly inhibited, and finally upregulate again. We believe that these findings provide valuable insights into potential genetic resources for enhancing the heat tolerance of *C. goeringii* and other orchids.

## Data Availability

The original contributions presented in the study are included in the article/Supplementary Material, further inquiries can be directed to the corresponding author.

## Supplementary Materials

The following supporting information can be downloaded at: https://www.mdpi.com/article/10.3390/genes15070915/s1, Table S1: physicochemical properties of GATAs.
